# Botulinum toxin A modifies nociceptive withdrawal reflex in subacute stroke patients

**DOI:** 10.1002/brb3.1162

**Published:** 2018-12-26

**Authors:** Elena Alvisi, Mariano Serrao, Carmela Conte, Enrico Alfonsi, Cristina Tassorelli, Paolo Prunetti, Silvano Cristina, Armando Perrotta, Francesco Pierelli, Giorgio Sandrini

In Alvisi et al. (2018), the present affiliations of the following authors will be added:

- Cristina Tassorelli, Silvano Cristina, and Giorgio Sandrini:
  Department of Neurorehabilitation, IRCCS Mondino Foundation, Pavia, Italy
- Armando Perrotta and Francesco Pierelli
  IRCSS fondazione Don Carlo Gnocchi, Rome, Italy
